# Antifungal Efficacy of Marine Macroalgae against Fungal Isolates from Bronchial Asthmatic Cases

**DOI:** 10.3390/molecules23113032

**Published:** 2018-11-20

**Authors:** Suresh Mickymaray, Wael Alturaiki

**Affiliations:** 1 Department of Biology, Central Bioscience Research Laboratories (CBRL), College of Science, Al-Zulfi-, Majmaah University, Majmaah 11952, Riyadh Region, Saudi Arabia; 2 Department of Medical Laboratories, College of Applied Medical Sciences, Majmaah University, Majmaah 11952, Riyadh Region, Saudi Arabia; w.alturaiki@mu.edu.sa

**Keywords:** bronchial asthma, fungal isolates, macroalgae, ethanolic fractions, fungicidal actions

## Abstract

Fungal sensitization is very common in bronchial asthmatic cases, and the connection with airway colonization by fungi remains uncertain. Antifungal therapy failure is a significant fraction of the cost and morbidity and mortality in the majority of the asthmatic cases. Hence, the present study aimed to investigate the antifungal activity of five marine macroalgae—*Acanthaophora specifera*, *Cladophoropsis* sp., *Laurencia paniculata*, *Tydemania* sp., and *Ulva prolifera*—which were tested on selected fungal pathogens isolated from 15 sputum of 45 bronchial asthmatic patients. The highest antifungal activity was observed in ethanol fractions of *L. paniculata* followed by *U. prolifera*, *Cladophoropsis* sp., *A. specifera*, and *Tydemania* sp. The minimum fungicidal concentration and minimum inhibitory concentration values of the ethanolic fractions of algal species were found to be 125–1000 µg/mL and 125–500 µg/mL, respectively. The algal extracts contained terpene alcohol, diterpene, steroids, sesquiterpene, and sesquiterpene alcohol, as determined by GC–MS/MS analyses. The present study shows that the marine macroalgae containing bioactive compounds had excellent inhibitory activity against a variety of fungal pathogens, which may be useful for combating fungal infections and recovering from chronic asthmatic states.

## 1. Introduction

Bronchial asthma is an inflammatory illness of the airways with characterized hypersensitiveness that could be deteriorated by several factors. It is a serious public health problem, affecting people of all ages with characterized symptoms of chest tightness, dyspnea, cough, and wheeze [1]. A common cause is consistent exposure to allergens, of which fungi play a substantial role. Fungal sensitization is very common in bronchial asthmatic cases, and the connection with airway colonization by fungi remains uncertain. Antifungal therapeutic failures increase the cost of treatment, morbidity, and mortality in over 80% of asthmatic cases in low and lower middle-income countries [2,3]. Various studies associated with antimicrobial resistance (AMR) predict that the death toll owing to AMR may exceed 10 million by 2050, potentially leading to greater mortality when compared with various malignancies [4,5,6]. The resistance of pathogenic fungi to existing antibiotics has become a global epidemic. Therapeutic drugs of choices for invasive fungal infections are very minimal when compared to therapeutic choices for bacterial infections [7]. In clinical practice, only three classes of antifungal drugs are available, i.e., polyenes, azoles, and echinocandins. In the last 30 years of therapeutic research, only a few antifungal drugs were elucidated [6]. Traditional plant-based medicine and bioactive natural products are being consumed as therapeutic medicine to enhance the prevailing treatments with fewer side effects [8].

Marine macroalgae are macroscopic, multicellular organisms, commonly known as seaweeds and classified as green algae (Chlorophyta), red algae (Rhodophyta), and brown algae (Phaeophyta). The majority of the algal species produce novel secondary metabolites due to extreme climatic and environmental stress such as salinity, light, temperature, and marine chemical diversity. They are a prospective source for structurally unique secondary metabolites with a wide range of biological activities such as antimicrobial [9], antiviral [10], antioxidant [11], anticancer [12], and anti-inflammatory [13] properties. Furthermore, they were reported for various bioactive compounds including alkaloids, polysaccharides, terpenes, phlorotannins, sterols, quinones, carbohydrates, proteins, minerals, lipids, polyunsaturated fatty acids, polyphenols, tocopherols, vitamin C, amino acids, carotenoids, chlorophylls, and phycobilins [14,15]. The Red Sea coast has an extensive coastline with an immense diversity of marine algae. *Acanthophora spicifera* and *Laurencia paniculata* are species of marine red algae in the class Florideophyceae under the phylum Rhodophyta. *Cladophoropsis* sp., *Tydemania* sp., and *Ulva prolifera* (synonymous with *Enteromorpha prolifera*) are species of marine green algae in the class Ulvophyceae under the phylum Chlorophyta. They are abundant in the Red Sea coast of Yanbu, Kingdom of Saudi Arabia throughout the year. According to previous reports in the literature, the antibacterial activity of marine macroalgae was extensively studied [16,17,18,19]. However, few reports are available on the antifungal activity of marine macroalgal species. Hence, the present study aimed to investigate the antifungal activity of marine macroalgae collected from the Red Sea coast against fungal pathogens isolated from sputum of moderately severe chronic bronchial asthmatic patients, who seek medical attention at Zulfi General Hospital, Kingdom of Saudi Arabia.

## 2. Results

**Figure 1:**
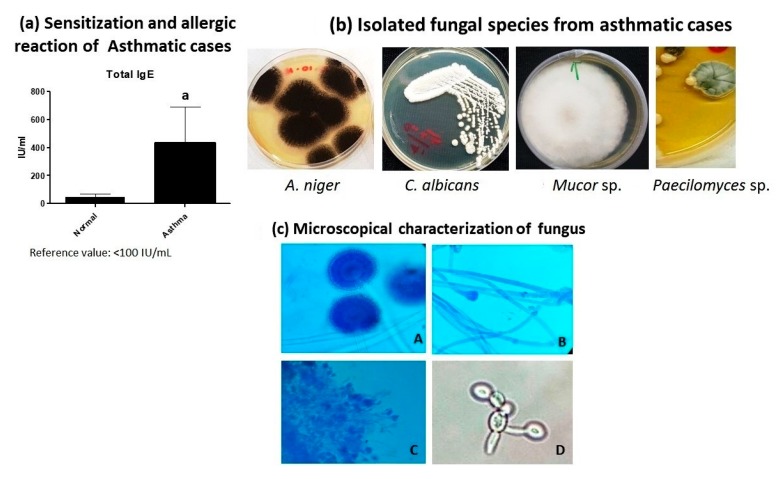
(**a**) Total immunoglobulin E (IgE), sensitization, and allergic reactions of asthmatic cases; (**b**) isolated fungal species from asthmatic cases; (**c**) microscopical characterization of fungus with lactophenol cotton blue mount: A, *Aspergillus niger*; B, aseptate hyphae and young conidiophores of *Mucor* sp; C, *Paecilomyces* sp.; D, germination tube test positivity of *Candida albicans.* Results are means ± SD. The letter a above the average bar denotes a significant difference with the normal at *p* < 0.05 using Tukey’s test.

Immunoglobulin E (IgE) estimation confirmed the sensitization and allergic reactions of chronic asthmatic cases. The levels of IgE detected in the patients ranged from 200–430 IU/mL, with 100 IU/mL considered as a reference value (Figure 1a). About 18 fungal pathogens were isolated from 15 cases out of 45 samples processed. The isolated fungi were identified as follows: *Candida albicans* (*n* = 11, 61.11%), *Aspergillus niger* (*n* = 4, 22.22%), *Mucor* species (*n* = 1, 5.55%), *Paecilomyces* species (*n* = 1, 5.55%), and unknown filamentous fungi (*n* = 1, 5.55%). The four major fungal isolates—*Candida albicans, Aspergillus niger, Mucor* species*,* and *Paeciliomyces* species—were taken as representative organisms for the antifungal study against algal extracts (Figure 1b). The filamentous fungi were identified based on colony morphology followed by lactophenol cotton blue mount, as well as a germination tube test (Figure 1c). The results of the antifungal activity of the studied five algal fractions are summarized in Table 1. The ethanolic fractions of *L. paniculata* and *U. prolifera* showed considerable antifungal activity with inhibition zone diameters varying from 16 to 17.6 mm at a concentration of 1000 µg/mL against all the tested organisms. Their antifungal activity was comparable to the standard antibiotic (amphotericin B, 100 units) used. The ethanolic fraction of *Cladophoropsis* sp. performed better in the inhibition of the growth of all kinds of tested fungi with a moderate zone of inhibition (10.6–11.6 mm). *A. spicifera* showed the lowest antifungal activity (10–10.6 mm) except for *Mucor* sp.; however, the ethanolic fractions of *Tydemania* sp. did not show any noticeable activity against the tested fungal pathogens except for *Mucor* sp. The ethanolic fractions of algal species were found to be the most active fractions with the exception of *Tydemania* sp., whereas petroleum ether fractions were not active on the tested pathogens.

The activity of the ethanolic fractions of five marine macroalgae—*A. spicifera, Cladophoropsis* sp., *L. paniculata*, *Tydemania* sp., and *U. prolifera*—against asthmatic fungal isolate was assessed using the agar well-diffusion method. The results were also compared with the growth inhibition of asthmatic fungal isolates by the standard antifungal drug (amphotericin B, 100 units) as indicated. Experiments were conducted in triplicate and results were expressed as means ± standard deviation. The superscript letters (a and b) above denote significant differences at *p* < 0.05 using Tukey’s test.

**Figure 2:**
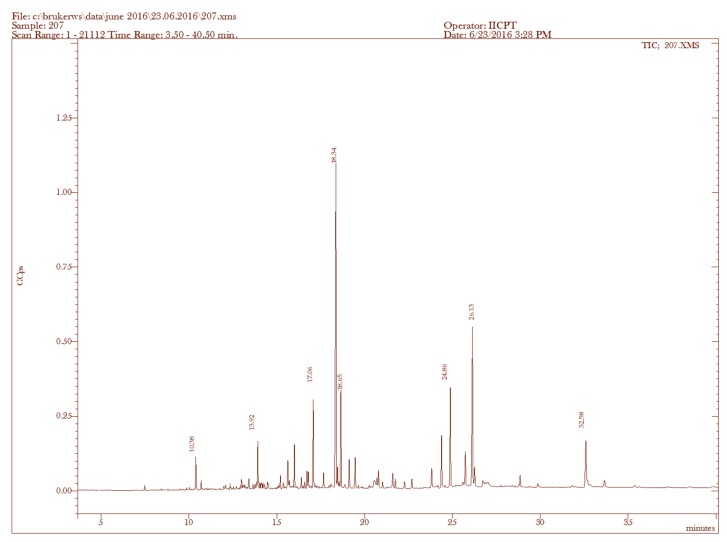
GC–MS/MS chromatogram formed by ethanolic fractions of *Laurencia paniculata*. The 436-GC Bruker model coupled with a triple quadrupole mass spectrometer was used to study the chemical composition of the ethanolic fractions of the two plants. The MS Workstation 8 and the library database of National Institute Standard and Technology (NIST Version 11.0) were used for the identification of the chemical components.

**Figure 3:**
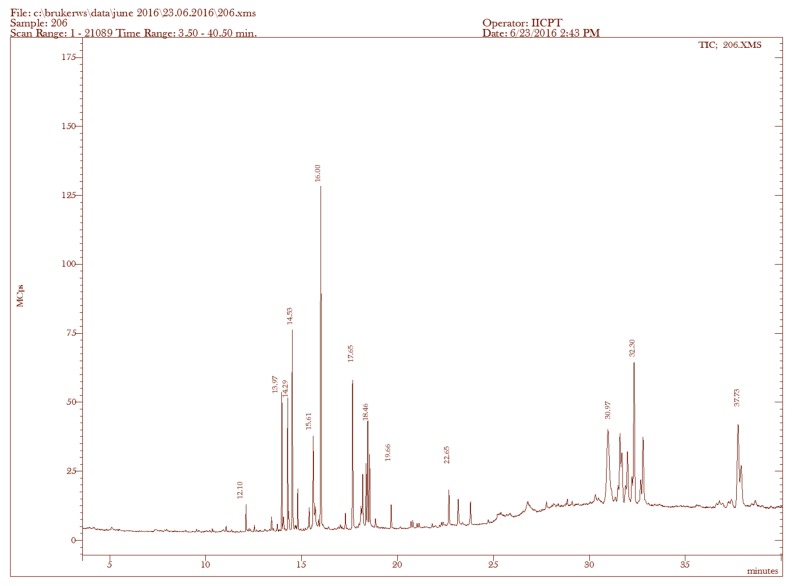
GC–MS/MS chromatogram formed by ethanolic fractions of *Ulva prolifera*. The 436-GC Bruker model coupled with a triple quadrupole mass spectrometer was used to study the chemical composition of the ethanolic fractions of the two plants. The MS Workstation 8 and the library database of National Institute Standard and Technology (NIST Version 11.0) were used for the identification of the chemical components.

Considering the superior antifungal potential of the ethanolic fractions of *L. paniculata* and *U. prolifera*, they were preferred for minimum inhibitory concentration (MIC) and minimum fungicidal concentration (MFC) analysis. The MIC and MFC values with fungicidal ratios of ethanolic fractions of algal species are shown in Table 2. Both MIC and MFC analyses on ethanolic fractions of *L. paniculata* and *U. prolifera* revealed the lowest value of 125 μg/mL with a fungicidal ratio of 1:1 in *C. albicans.* The fungicidal effect of ethanolic fractions of *L. paniculata* and *U. prolifera* was due to their MFC/MIC ratio, which was between 1:1 and 1:2. The spectral analysis of ethanolic fractions of *L. paniculata* and *U. prolifera* are shown in Figure 2 and Figure 3. In total, 33 and 23 prominent peaks were obtained in the ethanolic fractions of *L. paniculata* and *U. prolifera,* respectively. Among them, the 12 and 10 identified antimicrobial compounds, respectively are given in Table 3 and Table 4. There were three common antimicrobial compounds, i.e., 3,7,11,15-tetramethyl-2-hexadecene-1-ol, phytol, and dasycarpidan-1-methanol acetate (ester), identified in both fractions. The 3,7,11,15-tetramethyl-2-hexadecene-1-ol (7.32%) and cholestan-3-ol 2-methylene-(3β,5α) (13.91%) compounds are the major antimicrobial contents present in the ethanolic fractions of *L. paniculata* and *U. prolifera*, respectively. The biological activities listed in the tables are based on Dr. Duke’s Phytochemical and Ethnobotanical Databases created by Dr. Jim Duke of the Agricultural Research Service, United States Department of Agriculture (USDA).

## 3. Discussion

In the present study, ethanol and petroleum ether were used for the extraction of antifungal compounds from five selected macroalgal species. The ethanolic fractions of algal species exhibited superior antifungal activity, and petroleum ether fractions did not show any activity against the tested pathogens. Results obtained in the present study indicated that hydrophilic solvent fractions provided better activity. These results are in harmony with earlier reports [20,21,22,23,24,25]. In the present study, the species of Rhodomelaceae (*L. paniculata*) showed the strongest antifungal activity against the tested pathogens, which is in agreement with the findings of Rahelivao et al. [26]. They reported that the methanol extract of *L. complanata* exhibited antifungal activity against *C. albicans* with an inhibition zone of 11.5 mm. Previously, Alarif and his coworkers [27] isolated cholestane derivative 3⍺,6⍺-dihydroxy-5beta-cholestan-12-one and aldehyde derivative (E)-2-{(E) tridec-2-en-2-yl} heptadec-2-enal from *L. papillosa* collected from the Red Sea in Saudi Arabia, which showed considerable antifungal activity against *C. albicans*, *A. fumigatus*, and *A. flavus.*

In this study, an ethanolic fraction of *U. prolifera* exhibited inhibition zones (zone range: 16–17.3 mm) on tested fungi. The results agree with the findings of Chowdhury et al. [28], who reported that the growth of *C. albicans* was inhibited (15 mm) by an ethanolic extract of *Enteromorpha prolifera*. Correspondingly, the antifungal activity of *U. lactuca and U. fasciata* studied by Shobier et al. [29] against *Aspergillus flavipes* and *C. albicans* and found activities ranging from 10 to 32 mm. Additionally, Mashjoor et al. [30] demonstrated the antifungal activity in methanol and ethyl acetate extracts of *U.* *flexuosa* against *C. albicans*, and detected a moderate zone of inhibition (13 and 17 mm). Previously Ertürk and Taş [31] reported that *Ulva rigida* extracts showed activity against *A. niger* and *C. albicans* with 12 mm of inhibition zone. On the contrary, organic solvents fractions obtained from *U. lactuca* showed no activity against *C. albicans* reported by Guedes et al. [32]. In addition, *A. spicifera* had the lowest antifungal activity, in discrepancy with Pandian et al. [33] and Chowdhury et al. [28]. They reported that *A. spicifera* exhibited moderate antifungal activity against *A. niger, Microsporum gypseum,* and *C. albicans*.

Based on the broth dilution technique, the lowest concentration of ethanolic factions of *L. paniculata* and *U. prolifera* showed an MFC and MIC at 125 μg/mL against *C. albicans*. These results are in accordance with the findings of Shobier et al. [29], who reported that *U. fasciata* extract demonstrated an MFC and MIC at 155 and 128 μg/mL against *A. flavipes* and *C. albicans.* Similar work was done by Ertürk and Taş [31], who demonstrated that the antifungal activity of *U. rigida* extracts against *A. niger* and *C. albicans* had an MFC and MIC of more than 25 μg/mL and 10 μg/mL, respectively. Furthermore, a study by Genovese et al. [34] reported that the red algae *Asparagopsis taxiformis* showed antifungal activity against *A. fumigatus, A. terreus*, and *A. flavus* with a minimum inhibitory concentration range between 0.15 mg/mL and 5 mg/mL. From the results of the present study, it is clear that ethanol was the most effective solvent for the extraction of the bioactive compounds compared to petroleum ether. Furthermore, *L. paniculata* was the most effective marine algae against the five tested fungal species, followed by *U. prolifera, Cladophoropsis* sp., *A. specifera*, and *Tydemania* sp. Collectively, antifungal activity depends on the algal species, the efficiency of the extraction method, and the resistance characteristic of the tested fungi. The GC–MS/MS analysis showed various antimicrobial compounds present in ethanolic fractions of *L. paniculata* and *U. prolifera*. The two fractions contained a wide range of chemical classes, namely sesquiterpene, terpene, diterpene, steroid, etc. These compounds were already reported to have certain biological activities [29]. The genus *Laurencia* is a fruitful source of chemical diversity, including mainly sesquiterpenoids, diterpenoids, and terpenoids [35]. The identified sesquiterpenes were reported from various species of *Laurencia*, such as *L. tristicha* [36], *L. mariannensis* [37], *L. similis* [38], *L. saitoi* [39], and *L. okamurai* [40,41]*.* In this study, the ethanolic fractions of *U. prolifera* showed cholestan-3-ol, 2-methylene-(3β,5α) (13. 91%) as a major antimicrobial compound. Previous chemical investigation on *U. lactuca* methanolic extract revealed 42 compounds, of which the major components were 1,2-benzene dicarboxylic acid, bis(2-ethylhexyl) ester and palmitic acid, reported by Abbassy et al. [42]. Another study by Shobier et al. [29] found that the methanolic extracts of *U. fasciata* contained 17 compounds, and the prevailing compounds were palmitic acid, methylester, trichloromethyloxirane, and linolenic acid. The antifungal activity of *L. paniculata* and *U. prolifera* might be attributed to a wide range of chemical classes identified in the ethanolic fractions.

## 4. Materials and Methods

### 4.1. Collection of Macroalgae

**Figure 4:**
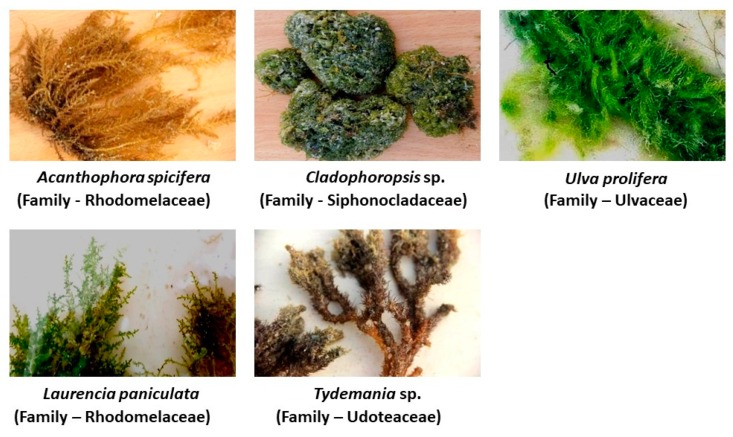
Aerial view of marine macroalgae collected from the rocky shore of Red sea coasts of Yanbu, Saudi Arabia.

Five macroalgae samples of *A. spicifera, Cladophoropsis* sp., *L. paniculata*, *Tydemania* sp., and *U. prolifera* were collected from the rocky shore of the Red Sea coast of Yanbu (24°24′39.5″ N; 37°26′49.2″ E and 24°03′52.25″ N; 38°05′05.1″ E), Saudi Arabia (Figure 4). Algal samples were preserved in sterile plastic bags and shipped to the laboratory under cold conditions at 4–8 °C.

### 4.2. Extraction of Bioactive Compounds from Algae Using Organic Solvents

About 5 kg of five different algal species, namely *A. spicifera, Cladophoropsis* sp., *L. paniculata*, *Tydemania* sp., and *U. prolifera* were harvested from the Red Sea coastal sampling site. The algal samples were separately rinsed with tap water and shade-dried, cut into small pieces, and powdered in a mixer grinder (MX1100XTX Hi-Power Electronic Keypad Blender with Timer, Toronto, OH, USA). Then, 500 g of a powdered sample of each algal species was extracted separately with 2 L of selected solvents, ethanol and petroleum ether*.* The algal powder was filled in the thimble and extracted to exhaustion with the solvents using a Soxhlet extractor (Thermo Fisher Scientific, Waltham, MA, USA) for 24 h. The obtained extracts were concentrated using a rotary flash evaporator (IKA RV 10, IKA -Werke GmbH & Co. KG, Darmstadt, Germany) at room temperature not exceeding 40 °C to get 45 g (*A. spicifera*), 37 g (*Cladophoropsis* sp.), 15 g (*L. paniculata*), 12 g (*Tydemania* sp.), and 8 g (*U. prolifera*) of the species. The fractions of algal samples were well preserved in airtight containers and kept at −20 °C for further analysis.

### 4.3. Collection of Fungal Pathogens

#### 4.3.1. Patients

In total, 45 sputum samples were obtained from chronic asthmatic (21 male and 24 female) patients with acute respiratory distress, featuring symptoms such as shortness of breath, cough, wheezing, chest tightness, and a few cases of excess mucus secretion. In addition, immunoglobulin E (IgE) was estimated as per the protocol of a human enzyme-linked immunosorbent assay (ELISA) kit (abcum, London, UK), and confirmed the allergic status of asthmatic cases. At the time of sampling, between May 2016 and April 2017, the specialty physicians at Al Zulfi General Hospital, Kingdom of Saudi Arabia, categorized all the patients as moderately severe asthmatics based on their clinical status. Written informed consent was obtained from all patients according to the protocols approved by the Majmaah University Ethical Committee (ID: MUREC-May.24/COM-2017/19). The minimum age of participants was 19 across both genders, and the maximum ages were 97 and 53 for female and male patients, respectively (average age: 39.62). All patients were on medication while the sputum specimen was collected; this medication included short-acting beta-agonists (SABAs) and a few cases with inhaled corticosteroids (ICS).

#### 4.3.2. Sputum Processing and Identification of Fungal Isolates

The expectorated samples were stored in ice, and the isolated mucus plug was inoculated within 2 h into two slants of Sabouraud dextrose agar containing 50 μg/mL chloramphenicol media using a class II biosafety cabinet. One was incubated at 37 °C and the other was incubated at 25 °C on the biological oxygen demand incubator for up to three weeks. Gram staining and germination tube test methods identified the grown tissue fungi (*C. albicans*). The filamentous fungi were determined using colony morphology, as well as lactophenol cotton blue mount.

#### 4.3.3. Determination of Antifungal Activity—Agar Well-Diffusion Method

The antifungal activity of the ethanol and petroleum ether fractions of macroalgal samples were evaluated using the agar well-diffusion method [43]. The inoculum used was prepared using the fungal pathogens from a 24-h culture on Sabouraud dextrose agar; a suspension was made in a sterile saline solution (0.85%). The turbidity of the suspension was adjusted with a spectrophotometer at 530 nm to obtain a final concentration to match that of a 0.5 McFarland standard (1–5 × 10^6^ cells/mL) [44]. The culture plates were prepared by pouring 20 mL of Sabouraud dextrose agar medium. The test fungal cultures were evenly spread over the media using a sterile cotton swab. Then, wells (6 mm) were made in the medium using a sterile cork borer, and 100 µL of the ethanol and petroleum ether fractions of macroalgal samples were transferred into separate wells. Then, these plates were incubated at 27 ± 2 °C for 48–72 h. After the incubation period, the results were observed, and the diameter of the inhibition zone around each well was measured. The formation of a clear inhibition zone around the well indicated the presence of antifungal activity. A standard antibiotic disc (amphotericin B, 100 units) was used as a control. All tests were performed in triplicate.

### 4.4. Minimum Inhibitory Concentrations (MICs)—Broth Dilution Method

#### Inoculum Preparation

The inoculum was prepared according to the method described in the Clinical and Laboratory Standards Institute CLSI M38-A protocol [45]. The inoculum of filamentous fungi is explained briefly. It was prepared by overlaying mature slants with sterile distilled water and gently scraping the surface with a wooden applicator stick. The suspension was permitted to sit for 5 min to allow large particles to settle down and then adjusted spectrophotometrically to the correct optical density for each species as outlined in M38-A, providing an inoculum concentration of 0.4–5 × 10^4^ conidia/mL, which was verified by colony count. The inoculum suspensions were diluted (1:50) in Roswell Park Memorial Institute (RPMI)-1640 medium buffered with 0.165 M morpholine propanesulfonic acid (34.54 g/L) at pH 7.0.

### 4.5. Determination of Minimal Fungicidal Concentration (MFC)

The MFC of each extract was determined by streaking 10 μL from the optically clear MIC well to the last growth well, and from the control onto Sabourad Dextrose agar [46]. The plates were incubated at 37 °C for four days. The MFC was determined as the lowest drug concentration at which no fungal colonies grew even after four days of incubation.

### 4.6. Antifungal Agents

Ethanolic fractions of two algal extracts of *L. paniculata* and *U. prolifera* were selected as antifungal agents after being condensed and dried using a Soxhlet apparatus method. To prepare, each was dissolved in sterile distilled water following the protocol of CLSI, and stock solutions of 5000 µg/mL were prepared, which were subsequently diluted in RPMI 1640 test medium for further various dilutions.

### 4.7. Preparation of Microdilution Plates and Antifungal Assay

Sterile disposable 96-well flat-bottom microdilution plastic plates with a normal capacity of approximately 300 µL were used; 100 µL from each of the tubes containing the corresponding concentration (2 × final concentration) of target algal extracts were dispensed into the wells in each column (from 1 to 10). For example, to column 1, the medium containing 2000 µg/mL was dispensed; to column 2, the medium containing 1000 µg/mL was dispensed. This pattern was continued to column 10, where the medium containing 4 µg/mL was dispensed. To each well of columns 11 and 12, 100 µL of RPMI-1640 medium was dispensed. Thus, each well in columns 1–10 contained 100 µL of twice the final algal extract concentrations (4 to 2000 µg/mL) in RPMI medium. Columns 11 and 12 contained double-strength RPMI-1640 medium, where one was used as a sterility control (100 µL of medium) and the other considerations were growth controls (100 µL of medium plus 100 µL of inoculum) [47]. The final well concentrations reached were 2 to 1000 µg/mL after addition of 100 µL of fungal inoculum to each well (columns 1 to 10). After the addition of inoculum, the microdilution plates were incubated at 35 °C for up to 48 h or until growth was visible in the sterility control well. Endpoint determination values were read visually with the aid of an inverted reading mirror. The MIC was defined as the lowest concentration that exhibited a 100% visual reduction in turbidity when compared with the control well at 48 h.

### 4.8. GC–MS/MS Analysis

The chemical composition of algal samples was investigated using GC–MS/MS in electron ionization mode. The GC–MS/MS was a Scion 436-GC Bruker model coupled with a triple quadrupole mass spectrometer with a fused silica capillary column BR-5MS (5% diphenyl/95% dimethylpolysiloxane) of length 30 m, internal diameter 0.25 mm, and thickness 0.25 μm. Helium gas (99.999%) was used as the carrier gas at a constant flow rate of 1 mL/min and an injection volume of 2 μL was employed (split ratio of 10:1). The column oven temperature program was as follows: 110 °C held for 3 min, up to 200 °C at 20 °C/min (not held), up to 280 °C at 5 °C/min (not held), up to 300 °C at 10 °C/min (held for 12 min). The injector temperature was 280 °C and the total GC running time was 40–50 min [48]. The mass spectrometer was operated in the positive electron ionization (EI) mode with an ionization energy of 70 eV. The solvent delay was 0–3.0 min. A scan interval of 0.5 seconds was used and fragments from *m*/*z* of 50 to 500 Da were programmed. The inlet temperature was set at 290 °C, while the source temperature was 250 °C. The relative percentage amount of each component was calculated by comparing its average peak area to the total areas. The software adapted to handle mass spectra and chromatograms was MS Workstation 8. The NIST Version 11.0 library database of National Institute Standard and Technology (NIST), containing more than 62,000 patterns, was used for identifying the chemical components. The spectra of the unknown components were compared with the spectra of known components stored in the NIST library.

### 4.9. Statistical Analysis

The data are given as means ± SD. The results were compared by one-way analysis of variance (ANOVA). Tukey’s test was used to identify significant differences among the means. Differences at the 5% level (*p* < 0.05) were considered statistically significant.

## 5. Conclusions

Bronchial asthma is a serious public health problem, affecting people of all ages with characterized symptoms. Exposure to fungal agents can worsen asthmatic symptoms. Antifungal therapeutic failures augmented the cost of treatment, morbidity, and mortality of most asthmatic cases. The present study shows that the selected marine macroalgae have excellent fungicidal activity against various fungal pathogens, which can assist recovery in chronic asthmatic states. In particular, the antifungal activity of *L. paniculata* was found to be excellent, and it can be recommended as a promising candidate for further fractionation to obtain novel antifungal substances. Future research investigating the synergistic effects of novel antifungal substances on major asthma mechanisms in vivo in an animal model of asthma is underway.

## Figures and Tables

**Table 1:** Antifungal activity of ethanolic factions of marine macroalgae.

Fungal Isolates	Ethanol Fractions: Zone of Inhibition (mm) with Standard Deviation for Triplicates (1000 µg/mL)					Standard antibiotic
	*Acanthophora spicifera*	*Cladophoropsis* sp.	*Laurencia paniculat*	*Tydemania* sp.	*Ulva prolifera*	(amphotericin B; 100 units)
*Aspergillus niger*	10.6 ± 1.15 ^a^	11.6 ± 0.57 ^a,b^	16.3 ± 0.57	-	16 ± 2	16.33 ± 0.57
*Candida albicans*	10 ± 2 ^a^	11 ± 2 ^a,b^	17.6 ± 0.57	-	17.3 ± 1.15	17 ± 1
*Mucor* sp.	-	10.6 ± 1.15 ^a^	17 ± 1	10 ± 1 ^a^	17 ± 1	17.33 ± 0.57
*Paecilomyces* sp.	10.6 ± 1.15 ^a^	11.3 ± 1.15 ^a^	17.3 ± 1.15 ^b^	-	16.6 ± 1.15	16.66 ± 0.57

- indicates fungal pathogens where no growth inhibition was observed.

**Table 2:** Minimum inhibitory concentration (MIC) and minimum fungicidal concentration (MFC) of ethanolic fractions of *L. paniculata* and *U. prolifera* with fungicidal ratio.

S.No	Fungal Pathogens	*L. paniculata*			*U. prolifera*		
		MIC μg/mL *	MFC μg/mL **	Fungicidal Ratio	MIC μg/mL *	MFC μg/mL **	Fungicidal Ratio
1.	*A. niger*	250	500	1:2	500	1000	1:2
2.	*C. albicans*	125	125	1:1	125	125	1:1
3.	*Mucor* sp.	250	500	1:1	500	1000	1:2
4	*Paecilomyces* sp.	250	500	1:2	500	500	1:1

* The Roswell Park Memorial Institute (RPMI) broth microdilution technique was applied to determine the MIC of the ethanolic fraction of *L. paniculata* and *U. prolifera* against the asthmatic fungal isolates. The MIC was defined as the lowest concentration that exhibited a 100% visual reduction in turbidity when compared with the control well at 48 h. ** The lowest algal extract concentration at which no fungal growth was identified even after four days of incubation.

**Table 3:** Bioactive compounds identified in ethanolic fractions of *L. paniculata* by GC–MS/MS analysis.

Sample	RT (min)	Name of the Compound	Molecular Formula	MW (g/mol)	Peak Area %	Compound Nature	** Activity
1.	7.47	(−)-Aristolene	C_15_H_24_	204	0.32	Sesquiterpene	Antibacterial, anti-inflammatory, fungicidal
2.	9.88	2-Naphthalenemethanol, decahydro-α,α,4a-trimethyl-8-methylene-, [2R-(2α,4aα,8aβ)]-	C_15_H_26_O	222	0.18	Sesquiterpene alcohol	Antibacterial, anti-inflammatory, fungicidal
3.	10.38	1-Naphthalenemethanol, 1,4,4a,5,6,7,8,8a-octahydro-2,5,5,8a-tetramethyl-	C_15_H_26_O	222	1.73	Sesquiterpene alcohol	Antibacterial, anti-inflammatory, fungicidal
4.	10.71	1,6,10-Dodecatrien-3-ol, 3,7,11-trimethyl-, (E)-	C_15_H_26_O	222	0.67	Sesquiterpene alcohol	Antibacterial, anti-inflammatory, fungicidal
5.	13.92	3,7,11,15-Tetramethyl-2-hexadecen-1-ol	C_20_H_40_O	296	7.32	Terpene alcohol	Antimicrobial, anti-inflammatory
6.	17.66	Phytol	C_20_H_40_O	296	2.03	Diterpene	Antimicrobial, anti-inflammatory Anticancer,
7.	18.65	Androst-5-en-17-one, 3-(acetyloxy)-19-hydroxy-, (3β)-	C_21_H_30_O_4_	346	5.98	Steroid	antimicrobial, anti-inflammatory Anticancer, antiasthma
8.	21.74	trans-Z-α-Bisabolene epoxide	C_15_H_24_O	220	1.35	Sesquiterpene alcohol	Anti-tumor, antibacterial, anti-inflammatory, fungicidal.
9.	22.69	Dasycarpidan-1-methanol, acetate (ester)	C_20_H_26_N_2_O_2_	326	0.76	Nitrogen compound	Antimicrobial
10.	28.85	Cholesta-3,5-diene	C_27_H_44_	368	0.50	Steroid	Antimicrobial, anti-inflammatory, anticancer, antiasthma
11.	32.58	Cholesterol	C_27_H_46_O	386	2.06	Steroid	Antimicrobial, anti-inflammatory, anticancer, antiasthma
12.	35.36	Cholest-4-en-3-one	C_27_H_44_O	384	0.05	Steroid	Antimicrobial, anti-inflammatory, anticancer, antiasthma

** The activity of the identified phytoconstituents was based on Dr. Duke’s Phytochemical and Ethnobotanical Databases. RT: retention time of the identified compounds; MW: molecular weight of the identified compounds.

**Table 4:** Bioactive compounds identified in ethanolic fractions of *U. prolifera* by GC–MS/MS analysis.

Sample	RT (min)	Name of the Compound	Molecular Formulae	MW (g/mol)	Peak Area %	Compound Nature	** Activity
1.	12.10	n-Heptadecanol-1	C_17_H_36_O	256	0.75	Alcoholic compound	Antimicrobial
2.	13.97	3,7,11,15-Tetramethyl-2-hexadecen-1-ol	C_20_H_40_O	296	3.38	Terpene alcohol	Antimicrobial, anti-inflammatory
3.	17.65	Phytol	C_20_H_40_O	296	5.28	Diterpene	Antimicrobial, anti-inflammatory, anticancer, diuretic
4.	22.65	Dasycarpidan-1-methanol acetate (ester)	C_20_H_26_N_2_O_2_	326	1.54	Nitrogen compound	Antimicrobial
5.	30.97	Cholestan-3-ol, 2-methylene-, (3β,5α)-	C_28_H_48_O	400	13.91	Steroid	Antimicrobial, anti-inflammatory, anticancer, antiasthma
6.	31.63	Stigmasta-5,22-dien-3-ol, acetate, (3β)-	C_31_H_50_O_2_	454	4.85	Steroid	Antimicrobial, anti-inflammatory, anticancer, antiasthma
7.	31.98	Pregn-5-en-20-one, 3-(acetyloxy)-17-hydroxy-, (3β)-	C_23_H_34_O_4_	374	5.24	Steroid	Antimicrobial, anti-inflammatory, anticancer, antiasthma
8.	32.30	Allopregn-5,16-diene-3β-ol-20-one acetate	C_23_H_32_O_3_	356	9.93	Steroid	Antimicrobial, anti-inflammatory, anticancer, antiasthma
9.	32.76	5,16,20-Pregnatriene-3beta,20-diol diacetate	C_25_H_34_O_4_	398	6.04	Steroid	Antimicrobial, anti-inflammatory, anticancer, antiasthma
10.	37.73	Stigmasta-5,24(28)-dien-3-ol, (3β,24Z)-	C_29_H_48_O	412	8.33	Steroid	Antimicrobial, anti-inflammatory, anticancer, antiasthma

** The activity of the identified phytoconstituents was based on Dr. Duke’s Phytochemical and Ethnobotanical Databases. RT: retention time of the identified compounds; MW: molecular weight of the identified compounds. The chemical composition of the ethanolic fraction of *L. paniculata* and *U. prolifera* was determined using gas chromatography (436-GC Bruker model) coupled with a triple quadrupole mass spectrometer.

## Abbreviations

µg	Microgram
AMR	Antimicrobial resistance
CLSI	Clinical and Laboratory Standards Institute
ELISA	Enzyme-linked immunosorbent assay
GC–MS/MS	Gas chromatography/tandem mass spectrometry
ICS	Inhaled corticosteroids
IgE	Immunoglobulin E
IU	International unit
MIC	Minimum inhibitory concentration
MFC	Minimum fungicidal concentration
mL	Milliliter
MW	Molecular weight
NIST	National Institute Standard and Technology
RPMI	Roswell Park Memorial Institute
RT	Retention time
SABA	Short-acting beta-agonist
UK	United Kingdom
USA	United States of America
